# Patterns of Screen Time From Ages 2 to 6–7 Years in South Brazil: A Prospective Study

**DOI:** 10.1111/cch.70033

**Published:** 2025-01-08

**Authors:** Otávio Amaral de Andrade Leão, Thaynã Ramos Flores, Daniela de Oliveira Nava, Pedro San Martin Soares, Joseph Murray, Marlos Rodrigues Domingues, Pedro Curi Hallal

**Affiliations:** ^1^ Department of Health and Kinesiology, College of Applied Health Sciences University of Illinois Urbana Champaign Champaign Illinois USA; ^2^ Postgraduate Program in Epidemiology Federal University of Pelotas Pelotas Brazil; ^3^ Medicine School Federal University of Pelotas Pelotas Brazil; ^4^ Postgraduate Program in Epidemiology and Human Development and Violence Research Centre (DOVE) Federal University of Pelotas Pelotas Brazil; ^5^ Postgraduate Program in Physical Education Federal University of Pelotas Pelotas Brazil

**Keywords:** children, cohort studies, preschool children, screen time, smartphone

## Abstract

**Background:**

Screen use has become nearly universal, especially in children. Therefore, it is important not only to comprehend its effects on health but also to understand its patterns of use. We aim to describe screen use patterns among children assessed at 2, 4, and 6–7 years, based on device, period of the day, and child/family characteristics.

**Methods:**

Longitudinal study, with participants of the 2015 Pelotas (Brazil) Birth Cohort, a population‐based study including all living newborns in the city of Pelotas between 1 January and 31 December 2015. Child/family characteristics used in the study were sex, skin color, family income, and maternal education. Screen time at 2 years was evaluated by TV time. At age 4, TV time and other screens (computer and videogames) was assesed. At 6–7 years, screen use was collected for each device (TV, smartphone, tablet/iPad, computer, and videogames).

**Results:**

At 2, 4, and 6–7 years, 1420, 3963, and 3857 had valid screen time data, respectively. Mean total screen time ranged from ~ 2.5 h per day at age 2 to ~ 5.5 h per day at age 6–7. At 2 years, no difference in screen time was found according to child/family characteristics. In general, boys presented higher screen time values at 4 and 6–7 years. No differences for ethnicity were observed. For family income and maternal education, the extreme groups presented higher use. Higher values of screen time were also observed during the evening and for children who did not attend school nor had home activities during the Covid‐19 pandemic.

**Conclusions:**

The results suggest that children are exceeding current screen time guidelines, with different patterns of use according to child/family characteristics. The high use of screens and more concentrated use during the evenings raise concern considering its possible negative effects on health.

**Summary**

- There was an increase in screen time from 2 to 6–7 years.
- The proportion of time spent on TV has decreased due to the increase in the use of other devices.
- Child screen time can be different considering socioeconomic and demographic characteristics.
- Screen use was higher during the evening, which can have specific impacts on health and learning outcomes.

## 1 Introduction

In the context of the modern world, personal screen use has evolved from being restricted to televisions to an almost ubiquitous use of pocket‐sized, mobile and portable devices across most countries. As a result of their portability, computers, tablets/iPads and especially smartphones have been incorporated into the routine of people from different social backgrounds and age groups, including children (Keeley and Little 2017). However, the escalation in early‐life screen use, spanning from birth to age 6 years, has given rise to significant concern (Dumuid 2020), primarily due to the rapid pace of brain development during this crucial period, the heightened susceptibility of the developing brain to early environmental influences (Grantham‐McGregor et al. 2007) and the growing abundance of media products designed for young children (Radesky and Christakis 2016).

The current World Health Organization (WHO) guidelines for screen time during early childhood suggest that children younger than 2 years should spend 0 min per day in sedentary screen time activities. For children of 2 to 5 years, the guidelines suggest no more than 1 h per day of sedentary screen time (World Health Organization 2019). As indicated by a recent meta‐analysis, only one in every four children younger than 2 years and one of every three children aged 2 to 5 years are meeting the WHO guidelines (World Health Organization 2019; McArthur et al. 2022). Another review showed that older children, 6 to 14 years, spent a mean of 2.77 h per day on screens, with nearly half meeting the guidelines (Qi, Yan, and Yin 2023). In addition, repeated population studies showed that there was an increase in around 1 h of screen time among children under five over the course of a decade (de Andrade Leão et al. 2023). This wide usage among young children has caused concern, especially considering possible effects of screen time on some health outcomes (Sanders et al. 2019; Stiglic and Viner 2019).

The coronavirus disease (COVID‐19) pandemic had a profound impact on various aspects of society, including the way children use screens and consume media. As a result of school closures and social distancing measures, there has been a significant increase in the use of technology by children for entertainment and education (Neville et al. 2022). According to a meta‐analysis that examined the duration of screen time in children, daily screen time significantly increased from 1.4 h before the pandemic to 2.7 h during the pandemic (Madigan et al. 2022). However, most of these studies of children's screen time were conducted in high‐income countries (Madigan et al. 2022).

In Brazil, the current screen time guidelines for children younger than 5‐years (da Silva et al. 2021) are almost the same as the WHO guidelines (World Health Organization 2019). Despite having its own guidelines, some studies have been showing higher screen time averages in young children, with around 1/3 meeting the current recommendations (de Andrade Leão et al. 2023; Rocha et al. 2021; Reis et al. 2024). Considering this, data from the 2015 Pelotas Cohort Study will provide an excellent opportunity to evaluate screen time over the early years, with the possibility to identify different patterns of use across childhood, in a population that was born in the digital media era in the Brazilian context.

To fully comprehend the effects of screen time on children's health, first, we need to understand how children spend their time on screens. There is a lack of detailed descriptive studies specifically aimed to understand screen time in children. Also, there is a need for studies describing the use by socioeconomic and demographic characteristics, types of screens, period of the day, in low‐ and middle‐income countries. Additionally, the use of longitudinal studies provides important insights on screen use over time during childhood. These descriptive studies contribute to create target guidelines and inform parents, educators, and public health professionals to better translate the theory into practice (Straker et al. 2018).

Thus, the objective of the present paper is to describe the screen use patterns among children assessed at ages 2, 4, and 6–7 years, based on device, period of the day, and child/family characteristics.

## 2 Methods

### 2.1 Study Population

Longitudinal data on children in the 2015 Pelotas Birth Cohort Study was used. In this study, all children born in Pelotas (a medium size city in Southern Brazil) between 1 January and 31 December 2015 and whose mothers resided in the urban area of the city, or Jardim America and Colonia Z3, were eligible to be enrolled in the cohort. During the perinatal study, at the time of delivery, 98.7% of the mothers were interviewed and provided information regarding prenatal care, socioeconomic, demographic, among others. Participants were invited for further follow‐up assessments at ages 3 months and 1, 2, 4 and 6–7 years (Hallal et al. 2018).

Specifically in this paper, we used data from birth, 2‐, 4‐ and 6‐ to 7‐year follow‐ups (Figure S1). In all those assessments, except from birth, children were evaluated in the university research clinic, with follow‐up rates higher than 90% (Murray et al. 2024). In addition to the cohort main objectives, to investigate early‐life exposures related to health, physical activity, and health inequalities, those follow‐ups also wanted to investigate life‐course health determinants over childhood, examine trends to compare with previous cohorts in the city, investigate psychosocial development and violence and understand the impacts of Covid‐19 (Murray et al. 2024).

The mothers or responsible of the participants signed the informed consent form before the beginning of any interview and data collection. Additional information on the logistics of the 2015 Pelotas (Brazil) Birth Cohort Study has been published elsewhere (Murray et al. 2024). All procedures were approved by the Federal University of Pelotas Research Ethics Committees under (0–4 years: School of Physical Education #26746414.5.0000.5313; 4 years and COVID‐19 pandemic follow‐ups: Faculty of Medicine #03837318.6.0000.5317 and #31179020.7.0000.5313; 6‐ to 7‐year follow‐up: 51789921.1.0000.5317).

### 2.2 Screen Time

At ages 2 and 4 years, the mother or caregiver reported the amount of time (hours + minutes) their child spent watching TV during the morning, afternoon, and evenings on a regular day. At age 4, similar questions regarding other screens (computer and videogames) were also applied. At 6–7 years, the collection instrument was more detailed, checking the screen time for each device (TV, smartphone, tablet/iPad, computer, and videogames) during the morning, afternoon, and evenings on a regular day. The details of each question used to assess screen time are included in Table S2.

### 2.3 Covariates

The following covariates were measured at child's birth: sex (female or male), maternal education in years (0–4, 5–8, 9–11, and 12 + years of schooling), and family income in minimum monthly wages (≤ 1, 1.1–3.0, 3.1–6.0, 6.1–10.0 and > 10.0). At the baseline, minimum monthly wage in Brazil was 788 reais (Brazilian currency)—or approximately $236.6 US dollars. At 4 years, skin colour of the child was registered by interviewer observation (black, white, and brown). It was also evaluated the child school status during the COVID‐19 pandemic (Do not attend school neither have home activities, attend school in person, attend school at home—online, and attend school in person and at home—hybrid).

### 2.4 Statistical Analyses

The analytical sample of the study comprised of children with screen time data at 2, 4 and 6–7 years, as described in the Figure S1. Analyses were conducted using Stata 16.0. Statistical significance was set at 5%. Proportions were used to describe sociodemographic variables in each follow‐up. A variable of total screen time was created using the sum of all screen time devices at each follow‐up. At 2‐years, only TV time was available and thus considered as total screen time for this follow‐up. Other screen time was considered as the sum of all screen devices except from TV. Differences in mean screen time according to device and maternal and child characteristics were examined using *t*‐tests or ANOVA. The prevalence of total screen time was categorized using the recommendations of the World Health Organization (WHO), according to age (0, ≤ 1 h, and ≤ 2 h) (World Health Organization 2019; Okely et al. 2022). A sensitivity analysis of screen time patterns and child school status during COVID‐19 pandemic was conducted using ANOVA, to see the influence of the pandemic on child screen behaviours.

## 3 Results

At 2, 4, and 6–7 years 4014, 4010, and 3867 were assessed. Of those, 1420, 3963, and 3857 had valid total screen time data at 2, 4, and 6–7 years. Characteristics of children are shown in Table 1. The distribution of sex, ethnicity, and family income was virtually the same for every follow‐up. The prevalence of children who followed the screen time guidelines at each age was 0.2%, 8.2%, and 15.0%, for 2, 4, and 6–7 years, respectively. Despite that, the prevalence of children who spent more than 2 h per day in screen based activities doubled from 2 to 6–7 years (42.5% to 84.5%).

**TABLE 1:** Cohort characteristics at baseline and 2‐, 4‐ and 6‐ to 7‐year follow‐up.

	2015 Cohort sample	2 years	4 years	6–7 years
	*N* (%)	*N* (%)	*N* (%)	*N* (%)
Total	4275 (100.0)	4014 (100.0)	4010 (100.0)	3867 (100.0)
Sex				
Female	2111 (49.4)	1984 (49.4)	1982 (49.4)	1914 (49.5)
Male	2164 (50.6)	2030 (50.6)	2028 (50.6)	1953 (50.5)
Ethnicity				
Black	386 (9.9)	378 (9.9)	386 (9.9)	374 (10.1)
White	2829 (72.5)	2764 (72.5)	2829 (72.5)	2679 (72.3)
Brown	685 (17.6)	670 (17.6)	685 (17.6)	653 (17.6)
Family income (minimum wage)				
≤ 1	498 (12.4)	463 (12.2)	461 (12.2)	448 (12.3)
1.1–3.0	1891 (47.1)	1787 (47.3)	1799 (47.7)	1754 (48.2)
3.1–6.0	1064 (26.5)	1006 (26.6)	1003 (26.6)	947 (26.1)
6.1–10.0	307 (7.6)	283 (7.5)	281 (7.5)	268 (7.4)
> 10.0	256 (6.4)	240 (6.4)	228 (6.0)	218 (6.0)
Maternal education (years)				
0–4	391 (9.2)	356 (8.9)	353 (8.8)	345 (8.9)
5–8	1095 (25.6)	1036 (25.8)	1044 (26.0)	1007 (26.1)
9–11	1458 (34.1)	1386 (34.5)	1394 (34.8)	1342 (34.7)
≥ 12	1330 (31.1)	1235 (30.8)	1218 (30.4)	1172 (30.3)
Prevalence of total screen time (hours)				
0	NA	3 (0.2)	7 (0.2)	111 (2.9)
0.1–1	NA	411 (28.9)	318 (8.0)	146 (3.8)
1.1–2	NA	403 (28.4)	657 (16.6)	342 (8.9)
> 2	NA	603 (42.5)	2981 (75.2)	3258 (84.5)

Abbreviation: NA, not available.

Table 2 presents the summary of screen time variables according to follow‐up and device. At age 2, mean total time was 149.5 min (~ 2 h30), 266.1 (~ 4 h30) min at age 4, and 333.8 (~ 5 h30) at 6–7 years. TV time rose from 2 to 4 years (149.5 to 171.9 min) but then remained stable until 6–7 years (173 min). The mean sum of other screen time at 4 years was 129.9 (~ 2 h) min and at 6–7 years it was 163.9 (~ 2 h30). At age 6–7, time on screens varied according to the device used. After TV, the highest use was of smartphones (131 min), followed by tablets/iPad (16.2 min), computer (8 min), and videogame (8.9 min).

**TABLE 2:** Mean screen time according to device and mother/child characteristics during 2‐ and 4‐year follow‐up.

	Total screen time^a^	TV time	Total other screens^b^
	Mean (SD)	Mean (SD)	Mean (SD)
2 years	NA	149.5 (120.3)	NA
Sex		*p* = 0.94	
Female	NA	149.7 (124.1)	NA
Male	NA	149.2 (116.7)	NA
Ethnicity		*p* = 0.58	
Black	NA	147.7 (113.8)	NA
White	NA	147.7 (119.5)	NA
Brown	NA	157.0 (131.0)	NA
Family income		*p* = 0.07	
≤ 1	NA	135.9 (113.5)	NA
1.1–3.0	NA	157.5 (130.7)	NA
3.1–6.0	NA	137.7 (106.9)	NA
6.1–10.0	NA	142.5 (111.2)	NA
> 10.0	NA	150.4 (90.8)	NA
Maternal education (years)		*p* = 0.31	
0–4	NA	133.9 (131.4)	NA
5–8	NA	155.3 (131.1)	NA
9–11	NA	152.5 (120.4)	NA
≥ 12	NA	145.5 (107.7)	NA
**4 years**	266.1 (178.5)	171.9 (127.3)	129.9 (117.5)
Sex	***p* = 0.01**	***p* = 0.02**	*p* = 0.39
Female	258.5 (172.7)	167.2 (123.2)	128.1 (117.9)
Male	273.5 (183.8)	176.4 (130.9)	131.7 (117.2)
Ethnicity	*p* = 0.29	*p* = 0.45	***p* = 0.01**
Black	266.8 (190.3)	172.0 (129.2)	139.4 (129.7)
White	264.3 (175.8)	170.8 (125.4)	126.5 (114.9)
Brown	276.3 (184.5)	177.9 (134.3)	141.2 (123.7)
Family income	***p* < 0.001**	***p* < 0.001**	***p* < 0.001**
≤ 1	264.7 (185.0)	175.9 (133.2)	144.3 (125.5)
1.1–3.0	283.3 (188.3)	181.4 (135.6)	139.9 (127.6)
3.1–6.0	258.2 (172.0)	165.5 (122.6)	122.2 (103.9)
6.1–10.0	229.2 (147.3)	149.9 (99.0)	104.7 (94.7)
> 10.0	202.8 (114.7)	137.7 (83.2)	87.5 (68.1)
Maternal education (years)	***p* < 0.001**	***p* < 0.001**	***p* < 0.001**
0–4	277.6 (219.6)	172.3 (148.5)	166.1 (150.6)
5–8	284.7 (188.1)	185.6 (136.9)	145.3 (128.4)
9–11	282.3 (178.2)	180.8 (131.1)	134.0 (118.1)
≥ 12	228.5 (149.7)	150.1 (103.0)	104.7 (91.5)

*Note:* Bolded *p*‐values are considered significant (< 0.05).

^a^ Total time: TV + Total other screens.

^b^ Total other screens: computer and videogames; NA: not available.

Screen time patterns according to child characteristics are shown in Table 2. At 2 years, no difference in mean screen time was found according to sex, ethnicity, or family income. At 4 years, TV and total screen time were higher among boys. Children categorized as brown had higher mean of other screens (141.2 min). Regarding family income, children in the lowest categories showed higher means for total screen time, TV, and total other screens.

At 6–7 years, boys presented significant higher means of screen time in all variables, except for tablet/iPad (Table 3). Higher means were also observed among black children for TV time (191.7 min) and higher mean videogame time for white children (10.3 min). At this age, children from the lower family income groups presented higher means for every screen time variable, except for tablet/iPad, computer, and videogame, which presented higher mean values for children with higher family income.

**TABLE 3:** Mean screen time according to device and mother/child characteristics for 6‐ to 7‐year follow‐up.

	Total time^a^	TV	Other screens				Total other screens^b^
			Smartphone	Tablet/iPad	Computer	Videogame	
	Mean (SD)	Mean (SD)	Mean (SD)	Mean (SD)	Mean (SD)	Mean (SD)	Mean (SD)
6–7 years	333.8 (209.1)	173.0 (140.8)	131.0 (137.2)	16.2 (61.7)	8.0 (44.4)	8.9 (36.6)	163.9 (161.4)
Sex	***p* < 0.001**	***p* < 0.001**	***p* = 0.01**	*p* = 0.55	***p* = 0.002**	***p* < 0.001**	***p* < 0.001**
Female	309.1 (198.3)	162.6 (134.4)	125.3 (128.6)	15.6 (60.3)	5.7 (36.0)	2.3 (16.9)	148.7 (148.6)
Male	358.0 (216.5)	183.2 (146.2)	136.7 (144.9)	16.7 (63.0)	10.2 (51.1)	15.4 (47.9)	178.8 (171.7)
Ethnicity	*p* = 0.23	*p* = 0.02	*p* = 0.24	*p* = 0.32	*p* = 0.30	*p* = 0.004	*p* = 0.95
Black	350.4 (220.4)	191.7 (155.8)	136.1 (142.2)	12.0 (52.5)	9.8 (55.4)	5.3 (28.7)	162.8 (171.3)
White	332.4 (201.8)	170.4 (136.7)	129.9 (134.4)	16.9 (60.6)	8.5 (44.5)	10.3 (39.8)	165.4 (158.0)
Brown	341.0 (228.4)	177.5 (144.0)	139.5 (147.3)	15.0 (70.5)	5.8 (40.6)	6.0 (26.8)	166.0 (173.6)
Family income	***p* < 0.001**	***p* < 0.001**	***p* < 0.001**	***p* < 0.001**	**p = 0.005**	***p* < 0.001**	***p* = 0.002**
≤ 1	310.9 (219.2)	163.5 (146.9)	128.5 (151.9)	13.7 (67.2)	5.8 (47.6)	2.8 (22.0)	150.5 (168.1)
1.1–3.0	351.6 (216.6)	184.1 (149.8)	144.1 (143.8)	12.0 (55.4)	6.0 (39.5)	7.6 (35.1)	169.7 (162.6)
3.1–6.0	341.7 (198.7)	170.2 (132.7)	133.3 (129.2)	18.5 (62.4)	12.2 (50.5)	10.5 (37.0)	174.3 (159.4)
6.1–10.0	295.2 (180.7)	155.2 (107.8)	100.9 (115.1)	29.5 (79.7)	5.1 (27.7)	14.7 (52.4)	150.1 (161.9)
> 10.0	275.8 (173.7)	138.6 (110.7)	81.7 (93.1)	29.3 (67.4)	9.3 (40.1)	17.3 (47.1)	137.2 (136.2)
Maternal education (years)	***p* = 0.002**	*p* = 0.07	***p* < 0.001**	***p* < 0.001**	***p* = 0.01**	***p* < 0.001**	***p* = 0.04**
0–4	325.2 (226.9)	166.4 (155.1)	136.1 (157.3)	10.8 (57.7)	10.6 (65.6)	4.9 (33.3)	162.4 (183.1)
5–8	331.7 (215.3)	178.1 (151.8)	135.6 (145.2)	9.4 (49.0)	4.8 (30.2)	5.5 (28.1)	155.2 (155.3)
9–11	350.1 (210.6)	177.7 (141.5)	143.2 (136.1)	14.8 (63.0)	7.3 (45.4)	8.3 (36.5)	173.5 (159.1)
≥ 12	319.4 (195.1)	165.4 (125.1)	111.4 (122.0)	25.2 (69.5)	10.7 (45.6)	13.6 (43.2)	160.8 (161.9)

*Note:* Bolded *p*‐values are considered significant (< 0.05).

^a^ Total time: TV + other screens (smartphone, tablet/iPad, computer, and videogames).

^b^ Total other screens: smartphone + tablet/iPad + computer + videogames.

**FIGURE 1:**
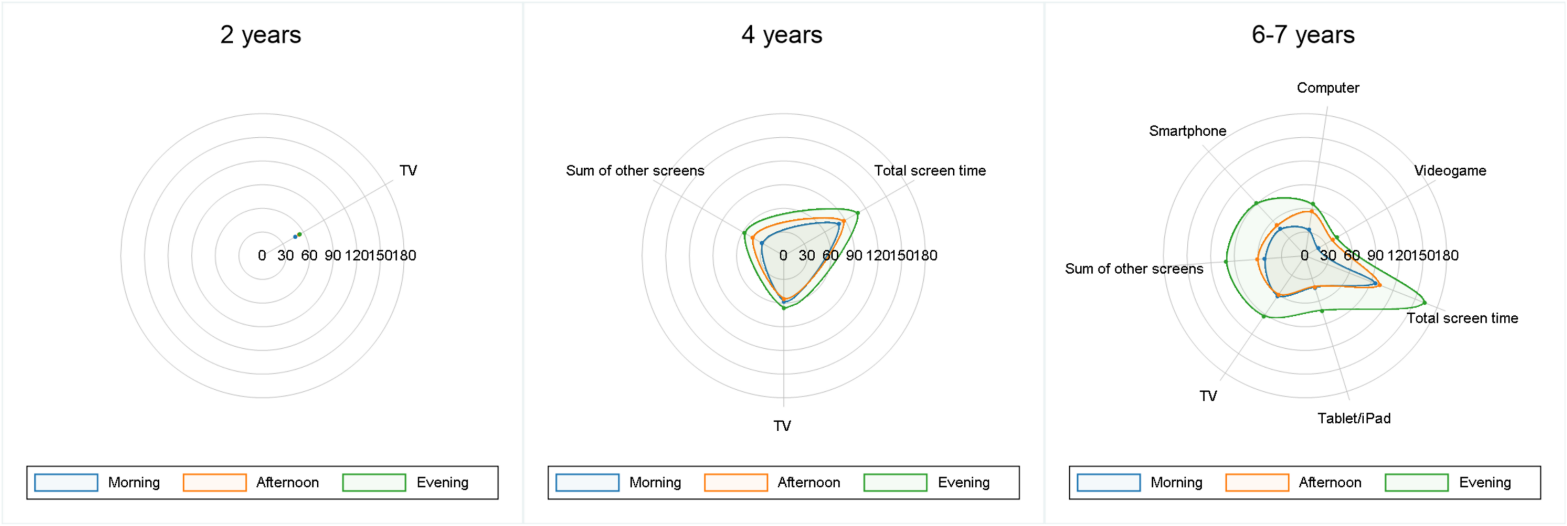
Mean screen time (in minutes) according to devices use throughout the day.

Figure 1 shows the distribution of screen time according to devices and use throughout the day. We can observe that at 2 years, there was a similar pattern of TV use. At 4 years, we can observe that children had higher means during the evening. At 6–7 years, children also showed higher means during the evening, except for computer and videogame.

**FIGURE 2:**
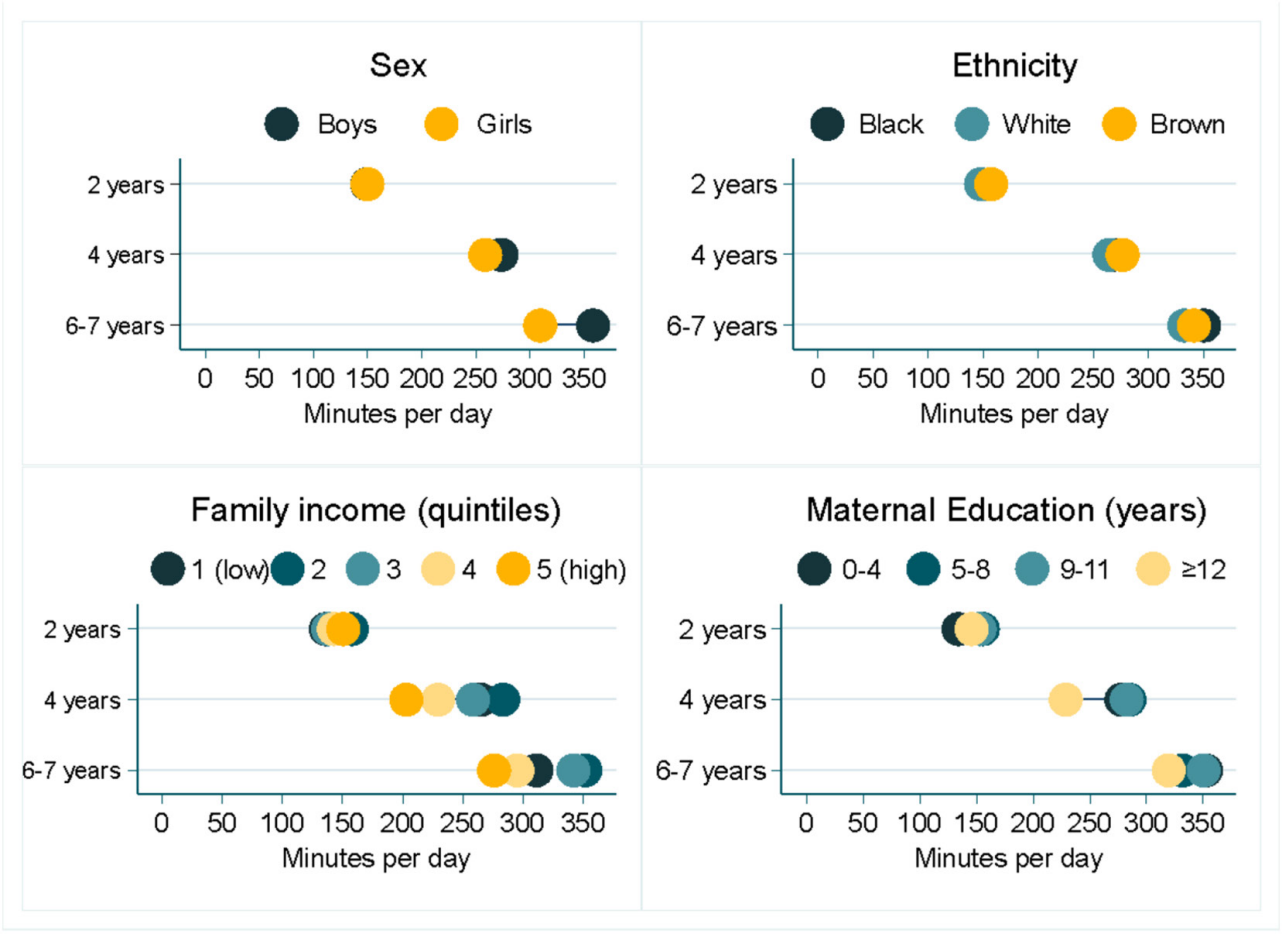
Equiplots of total screen time according to socioeconomic and demographic characteristics.

Figure 2 illustrates the different patterns of screen time according to the sample's characteristics. It can be observed that, at 2 years, mean screen time was very similar in all groups. For ethnicity, we also see similar means in all follow‐ups; however, for sex, starting at age 2, we can observe that boys started to present a higher mean compared to girls. For both family income and maternal education, we can observe that, at 4 and 6–7 years, children with higher family income and higher maternal education showed lower values of total screen time.

Results from Table S1 show the sensitivity analyses based on screen time during Covid‐19 pandemic. It can be observed that children who did not attend school in any form had higher values of total screen time, TV, smartphone, and total of other screens. For tablet/iPad and videogame, there were no significant differences; however, in computer use, children who had hybrid education showed significantly higher values.

## 4 Discussion

The present paper aimed to describe the patterns of children's screen time at ages 2, 4, and 6–7 years, differentiating by device, period of the day, and socioeconomic and demographic characteristics. Overall, we found that children mean screen time was high in all follow‐ups, with the pattern of use changing from TV to smartphone, and with higher use among boys, from intermediate groups of both family income and maternal education. We also observed higher use during the evening.

Mean total screen time varied from 149.5 min (~ 2.5 h) at age 2 to 333.8 min (~ 5.5 h) at age 6–7 years. The literature shows heterogeneous results, depending on the age of children and especially which devices were included in the screen time assessment, hindering comparison (Qi, Yan, and Yin 2023). It is also important to note that the data at 6–7 years were collected during the later stages of the Covid‐19 pandemic, which changed screen time behavior among children (Hedderson et al. 2023). If we consider that children spent approximately 8 h asleep per day (data not shown), children in this cohort spent approximately 30% of their waking time on screens at 4 and 6–7 years, which may limit their time performing other activities.

When comparing the prevalence of meeting the WHO guidelines for screen use at each age, we found that only 0.2% met the guidelines at age 2, 8.2% at age 4, and 15.5% at age 6–7 years. A meta‐analysis investigated the global prevalence of children under five who met the WHO screen time recommendations and found that 24.7% of children younger than 2 years met the recommendation, 35.6% of children aged 2 to 5 years met the recommendation of 1 h per day and 56% met the recommendation of 2 h per day (the same recommendation as children older than 5 years) (World Health Organization 2019; McArthur et al. 2022; Okely et al. 2022). Additionally, the prevalence of meeting the guidelines in the study was lower for all ages when compared to another Brazilian study that found approximately 30% of children aged 0–60 months met the guidelines (Rocha et al. 2021). Those values indicate that the prevalence of children in Pelotas who met the recommendations is very low, creating an alert considering the important role that screen time plays in children's lives.

The pattern of screen use by different devices changed over the years. At first, the study did not even had information on other devices during the 2‐year assessment. At age 4, we had information on TV and a sum of other screens, and it was observed that TV time represented 65% of the total time on screens. In the last assessment, TV time represented 51.8% of the total time on screens, showing a relative reduction of use in this device. This reflects the additional high use of smartphone use at this age, representing 39.2% of total screen time and 79.9% of other screens. This change on devices used is shown in other studies, demonstrating that smartphones are now a consolidated feature of life among children and adolescents' behavior (Radesky et al. 2020). Although the use of other devices, especially smartphones, may indicate a more ‘active’ use of screens, there is still the need to evaluate the content and context of its use (de Andrade Leão et al. 2023; Sanders et al. 2019).

The results indicate that there were no differences in screen use according to child's characteristics at age 2; however, there were some marked differences at 4 and 6–7 years by child sex, family income, and maternal education. Boys consistently presented higher screen use than girls. This is different to the findings from a systematic review of screen time correlates in children under five, which found a consistent lack of association on screen time and sex (Veldman et al. 2023). Considering another study in the Brazilian context, there was no association with sex among children from 2 to 4 years (Nobre et al. 2021). These gender differences must be elucidated in future research, especially considering the known gender inequality in physical activity since childhood (Kretschmer et al. 2023; Cla 2019).

For maternal education and family income, children from the extreme categories presented lower means than children in the middle categories, both at ages 4 and 6–7 years. This might be explained in three ways. First, children from the lower categories have lower access to screens (Mollborn et al. 2022; Tandon et al. 2012). Second, children in the higher categories have lower values because, even though they have access to screen devices, they also have more access to other activities, like sports clubs, music lessons, and language classes (Mollborn et al. 2022). Third, children in middle‐class families have access to screens but not to other activities, therefore resulting in higher screen time for those children (Tooth, Moss, and Mishra 2021).

Screen time according to ethnicity was different for specific devices at 4 and 6–7 years. Black children had higher mean time using other screens at 4 years and more TV time 6–7 years, respectively. There was also higher use of videogames for white children at 6–7 years. Those patterns observed for ethnicity are in accordance with maternal education and family income, in which more privileged children have access to specific devices, like videogames, and less privileged children have more concentrated TV use, for example (Mollborn et al. 2022).

There was more screen use in the evening period, compared to morning or afternoon, especially at ages 4 and 6–7 years. This result raises concerns considering the likely impact of screen time on child sleep outcomes and evidence of evening screen time negatively associated with sleep outcomes (Stiglic and Viner 2019; Janssen et al. 2020).

Some limitations of the study must be recognized. First, at age 2, only TV time was assessed, and due to a programming error in the questionnaire, only children born between July and December of that year had data. Despite that, children with data were not different from children without data (data not shown). Second, at age 4, the use of other screens was collected, but there was no discrimination by devices. Additionally, all our information was obtained from maternal perception, instead of objectively measured, and the question regarding the use of other screens only included the examples of computers and videogame, not smartphone, or tablet/iPad, which might have caused underreporting of other screen use at this age. Despite these limitations, some strengths of the present study should be highlighted. Descriptive studies, using data from a middle‐income country with large socioeconomic inequalities are scarce in the literature. The evaluation of different screen devices provided a more complete scenario, which then allows a better understanding of the impact on health and on target populations for interventions (Veldman et al. 2023). Based on our experience, we strongly suggest that future studies should aim at new screen possibilities, as technology changes quickly (as the change from TV to smartphones), and also objective methods for screen time data collection, as provided by many smartphones could be incorporated. Finally, using data from a birth cohort provides the perfect opportunity to track screen use over time and its possible late outcomes on health.

## 5 Conclusion

The results of the study suggest that children from the 2015 Pelotas Birth Cohort are exceeding current screen time guidelines, with values ranging from ~ 2.5 h at age 2 to ~ 5.5 h at age 6–7 years and with a more concentrated use during the evenings. Additionally, TV time is losing space to smartphones, and higher use was observed in boys and children from middle socioeconomic positions. Monitoring screen time patterns among children at this age are important to understand how this modern behavior will impact health and development throughout the life course.

## Author Contributions

**Otávio Amaral de Andrade Leão:** conceptualization, formal analysis, methodology, writing – review and editing, writing – original draft. **Thaynã Ramos Flores:** conceptualization, methodology, writing – review and editing, writing – original draft. **Daniela de Oliveira Nava:** writing – review and editing, writing – original draft. **Pedro San Martin Soares:** writing – review and editing, writing – original draft. **Joseph Murray:** writing – review and editing, writing – original draft. **Marlos Rodrigues Domingues:** writing – review and editing, writing – original draft. **Pedro Curi Hallal:** conceptualization, methodology, writing – review and editing, writing – original draft.

## Ethics Statement

All procedures were approved by the Federal University of Pelotas Research Ethics Committees under (0–4 years: School of Physical Education #26746414.5.0000.5313; 4 years and COVID‐19 pandemic follow‐ups: Faculty of Medicine #03837318.6.0000.5317 and #31179020.7.0000.5313; 6‐ to 7‐year follow‐up: 51789921.1.0000.5317).

## Conflicts of Interest

The authors declare no conflicts of interest.

## Supporting information


**Figure S1.** Flowchart of the children from the 2015 Pelotas (Brazil) Birth Cohort included at 2‐, 4‐, and 7‐year follow‐ups and children with screen time data.

**Table S1.** Sensitivity analyses on the pattern of screen time use at the 6‐ to 7‐year follow‐up according to child school status during the COVID‐19 pandemic.

**Table S2.** Screen time questions included in the 2, 4, and 6‐ to 7‐year follow‐ups of the 2015 Pelotas (Brazil) Birth Cohort.

## Data Availability

The data used for this study are available from the corresponding author upon reasonable request.
