# Supplementary material for: Patterns of Screen Time From Ages 2 to 6–7 Years in South Brazil: A Prospective Study
Source: Child Care Health Dev. 2025 Jan 8;51(1):e70033. doi: 10.1111/cch.70033 (PMC11710921; doi:10.1111/cch.70033)
Supplement: Supplementary file 1 — Figure S1. Flowchart of the children from the 2015 Pelotas (Brazil) Birth Cohort included at 2‐, 4‐, and 7‐year follow‐ups and children with screen time data. [file CCH-51-e70033-s003.docx]

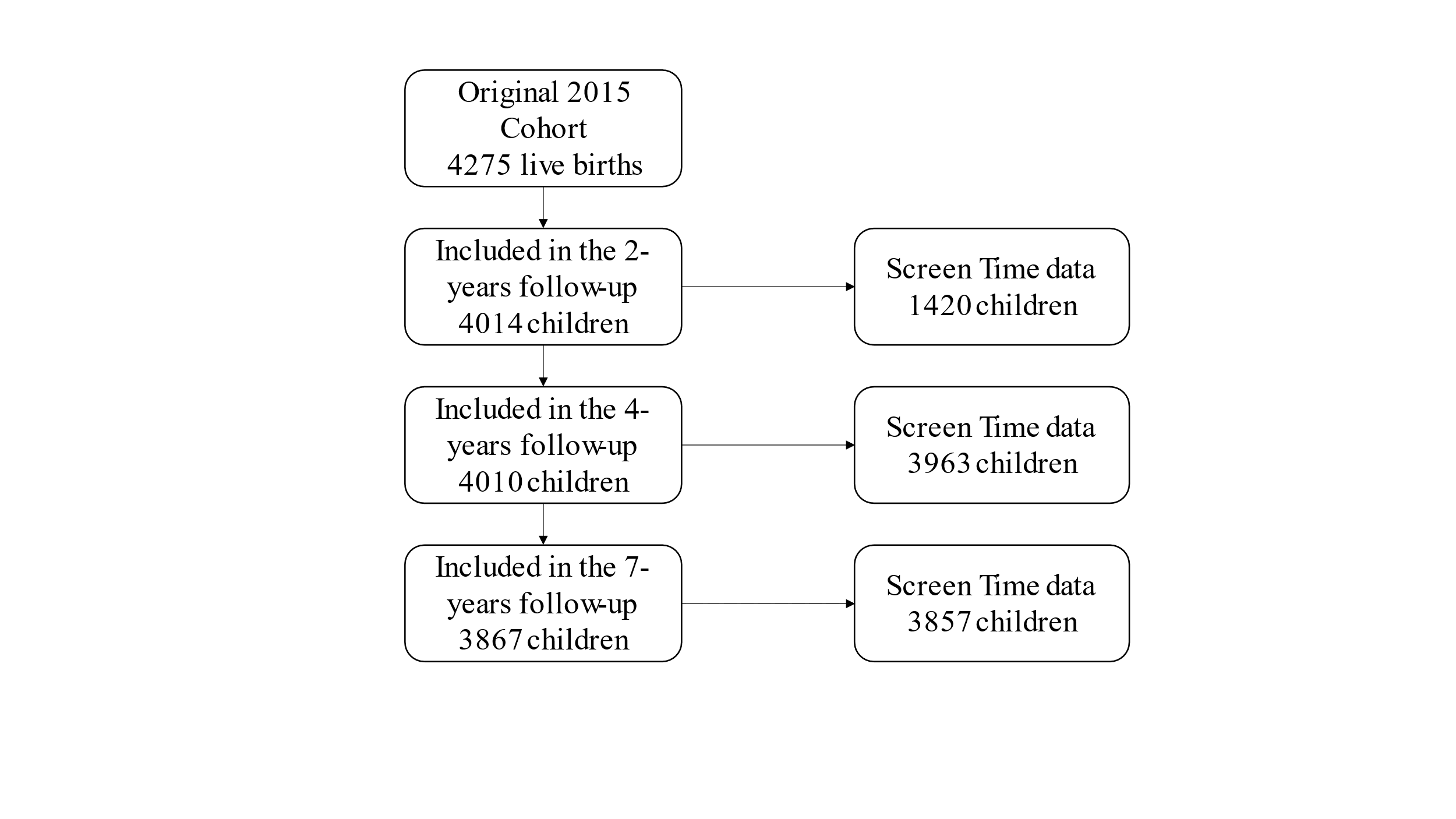


**Supplementary Figure 1.** Flowchart of the children from the 2015 Pelotas (Brazil) Birth Cohort included at 2, 4, and 7-years follow-ups and children with screen time data.
