# Supplementary material for: Patterns of Screen Time From Ages 2 to 6–7 Years in South Brazil: A Prospective Study
Source: Child Care Health Dev. 2025 Jan 8;51(1):e70033. doi: 10.1111/cch.70033 (PMC11710921; doi:10.1111/cch.70033)
Supplement: Supplementary file 2 — Table S1. Sensitivity analyses on the pattern of screen time use at the 6‐ to 7‐year follow‐up according to child school status during the COVID‐19 pandemic. [file CCH-51-e70033-s001.docx]

**Supplementary Table 1.** Sensitivity analyses on the pattern of screen time use at the 6-7 years follow-up according to child school status during the COVID-19 pandemic.

|  | **Total time#** | **TV** | **Other screens** | | | | **Total other screens*** |
| --- | --- | --- | --- | --- | --- | --- | --- |
|  |  |  | **Smartphone** | **Tablet/Ipad** | **Computer** | **Videogame** |  |
|  | **Mean (SD)** | **Mean (SD)** | **Mean (SD)** | **Mean (SD)** | **Mean (SD)** | **Mean (SD)** | **Mean (SD)** |
| **School status** | **p<0.001** | **p<0.001** | **p<0.001** | p=0.13 | **p=0.001** | p=0.15 | **p<0.001** |
| Do not attend school neither have home activities | 418.8 (257.3) | 219.4  (188.3) | 169.2 (188.4) | 20.9  (88.6) | 9.5  (59.8) | 12.8  (51.2) | 211.5  (224.6) |
| Attend school in person | 317.9 (201.2) | 166.2  (134.1) | 123.8 (130.0) | 14.9  (55.3) | 6.8  (37.6) | 8.2  (34.9) | 153.5  (150.4) |
| Attend school at home (online) | 388.0 (215.8) | 191.9  (153.1) | 157.8 (150.3) | 20.7  (82.7) | 10.4  (65.6) | 11.1  (42.2) | 200.1  (181.3) |
| Attend school in person and at home (hybrid) | 378.7 (215.8) | 191.1  (148.7) | 147.3 (143.2) | 20.5  (72.1) | 17.7  (62.1) | 10.6  (34.7) | 196.2  (179.3) |

#Total time: TV + other screens (smartphone, tablet/iPad, computer and videogames); *Total other screens: smartphone + tablet/iPad + computer + videogames.
