# Supplementary material for: Patterns of Screen Time From Ages 2 to 6–7 Years in South Brazil: A Prospective Study
Source: Child Care Health Dev. 2025 Jan 8;51(1):e70033. doi: 10.1111/cch.70033 (PMC11710921; doi:10.1111/cch.70033)
Supplement: Supplementary file 3 — Table S2. Screen time questions included in the 2, 4, and 6‐ to 7‐year follow‐ups of the 2015 Pelotas (Brazil) Birth Cohort. [file CCH-51-e70033-s002.docx]

**Supplementary Table 2.** Screen time questions included in the 2, 4, and 6-7 years follow-ups of the 2015 Pelotas (Brazil) Birth Cohort.

| **2 Years** | |
| --- | --- |
| The child watches television? | no 0  yes 1  IGN 9 |
| How much time the child watches television in the morning? | __ __ hours  __ __ minutes |
| How much time the child watches television in the afternoon? | __ __ hours  __ __ minutes |
| How much time the child watches television in the evening? | __ __ hours  __ __ minutes |
| **4 Years** | |
| The child watches television? | no 0  yes 1  TV Always on 2  IGN 9 |
| How much time the child watches television in the morning? | __ __ __ minutes |
| How much time the child watches television in the afternoon? | __ __ __ minutes |
| How much time the child watches television in the evening? | __ __ __ minutes |
| The child use computer, tablet/Ipad, videogame or cellphone/smartphone? | no 0  yes 1  IGN 9 |
| How much time the child use computer, tablet/Ipad, videogame or cellphone/smartphone in the morning? | __ __ hours __ __ minutes |
| How much time the child use computer, tablet/Ipad, videogame or cellphone/smartphone in the afternoon? | __ __ hours __ __ minutes |
| How much time the child use computer, tablet/Ipad, videogame or cellphone/smartphone in the evening? | __ __ hours __ __ minutes |
| **6-7 Years** | |
| The child watches television in most days? | no 0  yes 1  TV Always on 2  IGN 9 |
| How much time the child watches television in the morning? | __ __ hours __ __ minutes |
| How much time the child watches television in the afternoon? | __ __ hours __ __ minutes |
| How much time the child watches television in the evening? | __ __ hours __ __ minutes |
| The child use computer in most days? | no 0  yes 1  IGN 9 |
| How much time the child use computer in the morning? | __ __ hours __ __ minutes |
| How much time the child use computer in the afternoon? | __ __ hours __ __ minutes |
| How much time the child use computer in the evening? | __ __ hours __ __ minutes |
| The child use cellphone/smartphone in most days? | no 0  yes 1  IGN 9 |
| How much time the child use cellphone/smartphone in the morning? | __ __ hours __ __ minutes |
| How much time the child use cellphone/smartphone in the afternoon? | __ __ hours __ __ minutes |
| How much time the child use cellphone/smartphone in the evening? | __ __ hours __ __ minutes |
| The child use tablet/iPad in most days? | no 0  yes 1  IGN 9 |
| How much time the child use tablet/iPad in the morning? | __ __ hours __ __ minutes |
| How much time the child use tablet/iPad in the afternoon? | __ __ hours __ __ minutes |
| How much time the child use tablet/iPad in the evening? | __ __ hours __ __ minutes |
| The child use videogame in most days? | no 0  yes 1  IGN 9 |
| How much time the child use videogame in the morning? | __ __ hours __ __ minutes |
| How much time the child use videogame in the afternoon? | __ __ hours __ __ minutes |
| How much time the child use videogame in the evening? | __ __ hours __ __ minutes |
